# Scaling laws for group-constrained, subject-specific task fMRI analyses

**DOI:** 10.64898/2026.07.22.740076

**Published:** 2026-08-15

**Authors:** Ruimin Gao, Anna A. Ivanova

## Abstract

Functional MRI provides a powerful way to characterize the architecture of the human brain. Yet individual brains often vary in the exact anatomic locations of functionally specific regions; as a result of this variability, traditional random-effects group analyses (RFX) often require unrealistically large sample sizes to converge onto stable results. Here, we explore the scaling patterns for an alternative fMRI analysis framework that accounts for inter-individual topographic variability while still capturing spatial similarities--group-constrained, subject-specific (GcSS) analysis. Using sixteen contrasts from four Human Connectome Project tasks (spanning language, social cognition, motor, and working memory), we first show that the GcSS approach yields much larger effect sizes than traditional RFX analyses. We then investigate the impact of sample size (N=10-450) on the estimation of three key GcSS outputs: (1) group probabilistic maps for the contrast of interest, (2) group-level parcels that serve as anatomical constraints for subsequent functional region of interest (fROI) definitions in individual subjects, and (3) effect sizes of fROI responses to task conditions. For most contrasts, the probabilistic maps show good reliability at N=100 (intraclass correlation, ICC=.75) and excellent reliability at N=200 (ICC=.90). Most group parcels also achieve substantial agreement at N=100 (Dice coefficient, DC=.80). Critically, once robust group parcels are established, the subject-specific portion of the analysis can proceed with much smaller sample sizes: even samples of N=10 participants yield accurate effect size estimates within subject-specific fROIs, detecting 80% of practically meaningful effects (effect size ≥ 0.2); with N=20, this increases to 90%. The scaling patterns we observed held not only in the cortex, but also in the cerebellum. Our results challenge the view that fMRI research requires large samples: once the broad region of interest is established (with N=100 or higher), fROI-based analyses can yield generalizable results with small sample sizes (N=10 or N=20).

## Full Text Availability

The license terms selected by the author(s) for this preprint version do not permit archiving in PMC. The full text is available from the preprint server.

